# Role of Built Environments on Physical Activity and Health Promotion: A Review and Policy Insights

**DOI:** 10.3389/fpubh.2022.950348

**Published:** 2022-07-12

**Authors:** Jingjing Zhong, Wenting Liu, Buqing Niu, Xiongbin Lin, Yanhua Deng

**Affiliations:** ^1^Department of Geography and Spatial Information Technology, Ningbo University, Ningbo, China; ^2^Ningbo Universities Collaborative Innovation Center for Land and Marine Spatial Utilization and Governance Research at Ningbo University, Ningbo, China; ^3^Zhiweibing Center, Ningbo Municipal Hospital of Traditional Chinese Medicine, Ningbo, China

**Keywords:** built environments, physical activity, public health, urban planning, review

## Abstract

As urbanization and motorization continue worldwide, various health issues have emerged as a burden between individuals, families and governments at all levels. Under the prevalence of chronic disease, this review synthesizes research on the impact of the various built environments on the multiple health outcomes from a methodological and mechanistic perspective. Besides, it attempts to provide useful planning and policy implications to promote physical activity and health benefits. The finds show that: (1) Current literature has used a variety of dataset, methods, and models to examine the built environment–health benefit connections from the perspective of physical activity; (2) The prevalence of chronic diseases is inextricably linked to the built environment, and policy interventions related to physical activity and physical and mental wellbeing of urban residents should be emphasized; (3) The impact of the built environment on health is manifested in the way various elements of the physical environment guide the lifestyle of residents, thereby influencing physical activity and travel; (4) Given the changes that have occurred in the built environment during the current urban expansion, the link between urban planning and the public health sector should be strengthened in the future, and the relevant authorities should actively pursue policies that promote urban public health in order to improve the health of residents. Finally, it proposes potential policy insights for urban planning and development toward a healthier city and society.

## Introduction

Public health is one of the most important concerns of governments, individuals, and researchers, and a variety of factors would affect an individual's physical or mental health conditions. The emerging infectious diseases (EID) have long been a threat to public health, social stability, and economic development. For instance, the severe acute respiratory syndrome (SARS) that was recognized in February 2003 has cumulatively caused 8,096 probable SARS cases (1). The epidemic of coronavirus disease (COVID-19) has caused worldwide 518,055,132 and 6,253,570 confirmed cases and confirmed deaths worldwide, respectively at the end of 10 May 2022 (https://coronavirus.jhu.edu/map.html) (2). Since human beings would often fail to be well-prepared for the new respiratory virus, how to better prevent the new respiratory virus is gradually becoming important. Individuals' health condition is one of the most significant factors that can influence the quality of life (QOL) or even the value of life (VOL). Under the large-scale urbanization and motorization in developed and developing countries, the health conditions would become worse and various health problems (e.g., the epidemic of obesity, diabetes, and cardiovascular disease) are becoming a heavy burden for individuals, families, and governments at different levels.

Among several factors that would influence health conditions, the role of built environments both at the community and city scale has drawn close attention due to its proactive positive effects with self-directed activities with relatively low costs. For instance, high-density land development would be associated with a high level of physical activity, which is an important element to promote individuals' health conditions (3). Exposures to (un)favorable environments are generally associated with physical activity, healthy habits, healthy conditions among different social groups (4). Urban planning and urban development have made considerable efforts to promote public health through spatial planning and policy interventions, such as the concept and implementation of the garden city, broad-acre city, healthy city (5–7). The core vision of these emerging urban planning paradigms is to plan and facilitate optimal built environments favoring economic efficiency, social connections, and public health, aiming at reaching a good level of quality of life.

Currently, there are still several research difficulties and gaps in the study of various built environments and the associated impacts on physical and mental health through a variety of influential pathways. First, the impacts of built environments would significantly vary on both physical and psychological health conditions, which would make it hard to better assess the multifaceted impacts of built environments at different scales ranging from building, street, and community to local and regional scales. Second, there are a variety of ways to measure the components of built environments as well as physical and psychological health situations. In addition, considering the impacts of different modeling methods, the connections between built environments and public health would be sensitive to various methods applied as well as the issue of modifiable area unit problem (MAUP). Third, in terms of the potential connection between built environments, physical activity, and public health, how to incorporate these results into urban planning and policy to promote sustainable and healthy communities and cities still need to be further studied.

Indeed, these positive health outcomes of the various built environments have also been criticized potentially due to the impacts of self-selection and the MAUP (8, 9). For instance, certain studies use cross-sectional data to examine the built environment—physical activity relationship would fail to account for neighborhood self-selection effect (10), which becomes one of the major limitations of evidence (11). Whether and how the built environment can improve health conditions remain to be systematically reviewed. In particular, various impacting pathways and complicated mechanism of action should be considered.

Physical inactivity is highly associated with chronic diseases, thus significantly reducing the quality of life and rising healthcare costs (12). A well-designed built environment that promotes physical activity would help reduce physical inactivity levels and promote public health. Individuals would have a broader demand, including justice in public health (13). The planning, construction, and operation of the built environment would heavily rely on government expenditure. As a result, a better understanding of the built environment and health connection can provide helpful implications for public policy. The objective of this research is to conduct a systematic literature review on the built environment and health connections and then propose potential new policy insights for urban planning and development toward a healthier city.

The structure of this study is organized as follows. Next section will present the global prevalence of chronic disease and its connections to the impacts of built environments. After that, it will discuss the connections between various built environments and their multiply health outcomes both from the methodology and mechanism perspective. Before the last section of conclusion and discussion, it attempts to provide certain helpful planning and policy implications to promote individuals' health benefits through the reshaping of current built environments both at the local and city scale.

## The Prevalence of Chronic Diseases

Chronic diseases and public health are affected by many direct or indirect factors such as individual physiological characteristics, lifestyle, and built environments. The rapid urbanization and industrialization, land expansion, as well as economy-oriented development mode, have brought about great changes in urban built environments, such as land-use patterns, transportation, and open spaces, which have a great impact on urban public health. On the one hand, the air pollution problems caused by rapid industrial development and the massive use of private vehicles have contributed to the spatial range and intensity of urban residents' exposure to polluted environments, largely leading to the increase of respiratory system and cardiovascular diseases. Outdoor air pollution has emerged as the fourth leading risk factor accounting for worldwide pre-mature deaths, which significantly reduce economic development, with an estimated 5.5 million lives and US$ 225 billion lost in 2013 globally (14).

On the other hand, the rapid urban expansion would decrease access to active transportation and indoor or outdoor activities, which would also lead to the prevalence of chronic diseases, such as for overweight, obesity, cardiovascular disease, and diabetes. The obesity epidemic is becoming one of the most serious global health challenges in the 21st century (15). According to the information released by the World Health Organization, overweight and obesity were recognized as one of the top 10 health risks (16), and 39% of adults (age ≥18) were overweight and 13% were obese in 2016, which was nearly triple than that in 1975 (17). As a country whose residents were considered to be lean, China also has experienced the continuing increase of overweight and obese populations in the past decades. Obesity-related chronic diseases are expected to be one of the leading causes of death in China.

**Figure 1:**
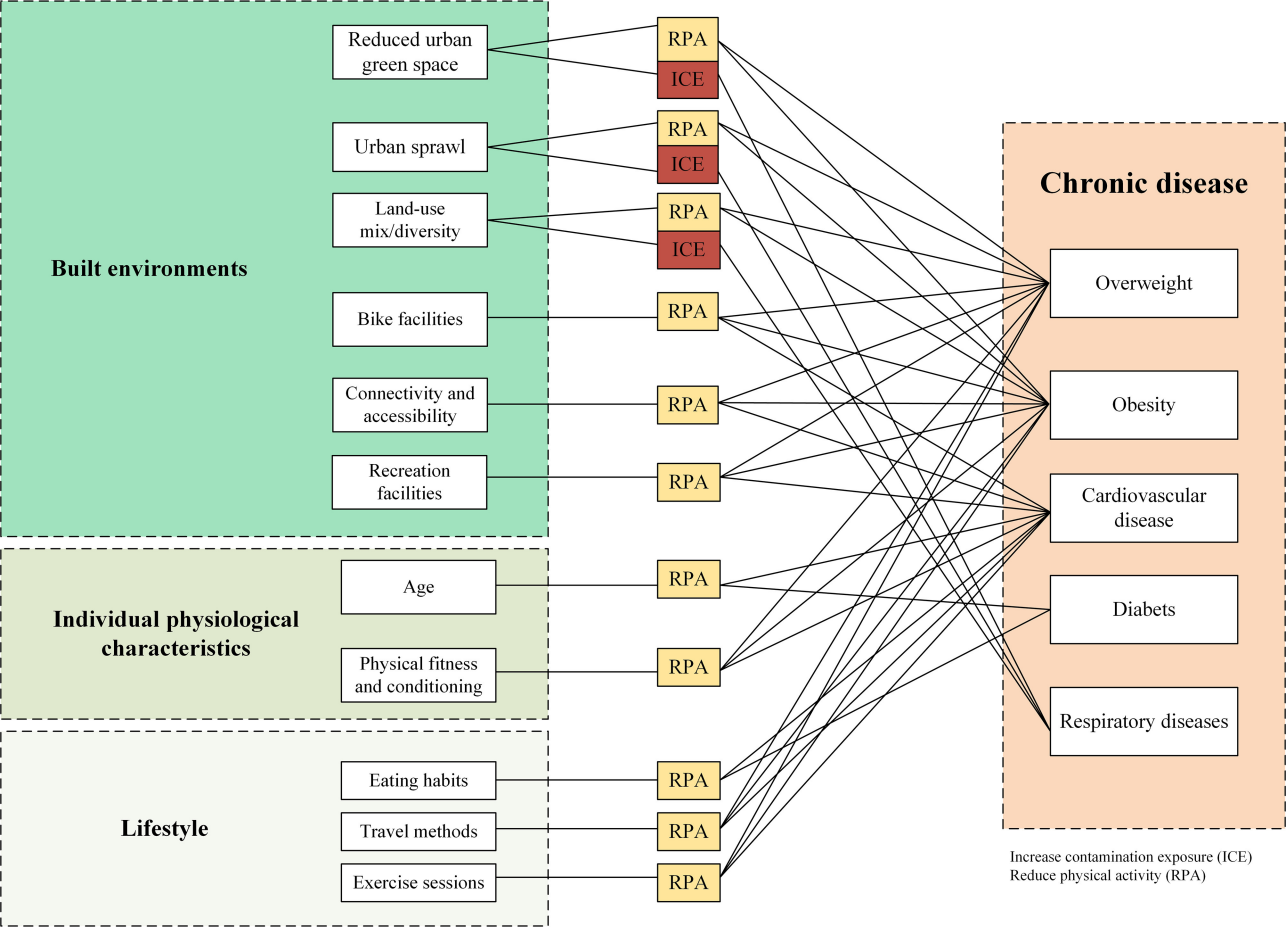
A variety of factors would influence chronic disease through exposure and physical activity.

At the macro level, there would remain potential pathways of the prevalence of chronic disease (such as for overweight, obesity, cardiovascular diseases, diabetes, and respiratory diseases) with a particular focus on factors related to various built environments, individual physiological characteristics, and lifestyle habits. The causes of major chronic diseases consist of two major factors: reduced physical activity and increased contamination exposure (Figure 1). Among the connections between built environments and public health, various complicated influential pathways of such connections are conceptualized under different social and spatial conditions and some of them are statistically significant or insignificant under the influences of contextual factors (such as particular features of individual, community, or urban) and methodology factors (such as dataset sample selection, and modeling methods). Indeed, current literature has tried to clarify such a kind of connection by adopting more appropriate data and models.

From the perspective of urban planning and development, it is increasingly important to optimize the spatial layout and distribution of built environment elements, including land-use, buildings, and transportation systems, to reduce pollution exposure and to guide residents to strengthen physical activity. To cope with the increasing chronic diseases, the built environment's impact on public health should be considered in advance during urban planning and construction stages, and the effective measures and guidelines should be formulated to help reduce the economic and social costs of health problems. Following this trend, it is urgent to pay attention to the influence mechanism of the built environment on physical activity as well as the physical and mental health of residents through effective planning or policy interventions.

## Connecting Built Environments and the Associated Health Outcomes

The concept of health has gradually evolved from the absence of disease into comprehensive good physical, social and psychological conditions (18). A large number of studies have shown that health condition is the result of multiple factors, which is not only affected by individual physiological characteristics and lifestyle, but also is closely related to urban land-use, development density, transportation system, and other built-up environments (19–21). In contrast to passive health promotion such as medicine and healthcare, built environmental planning and its optimization can encourage residents to actively participate in physical activity, reduce pollution exposure, and then enjoy the health benefits.

Under the rapid urbanization and economic development, the built environments have been significantly changed, such as green spaces, streets, and leisure infrastructure. For example, due to automobile-oriented development, the required land planned and constructed for motorization has experienced dramatic growth, at the sacrifice of the land designed for green transportation, such as walking and bicycle. The deficiency of and inaccessibility to infrastructure for physical activity and has shown significant impacts on individuals' health conditions. The considerable changes in the built environment can significantly affect individuals' physical and mental health conditions through their impacts on physical activity, air pollution exposure, and access to healthy food places or healthcare services.

**Figure 2:**
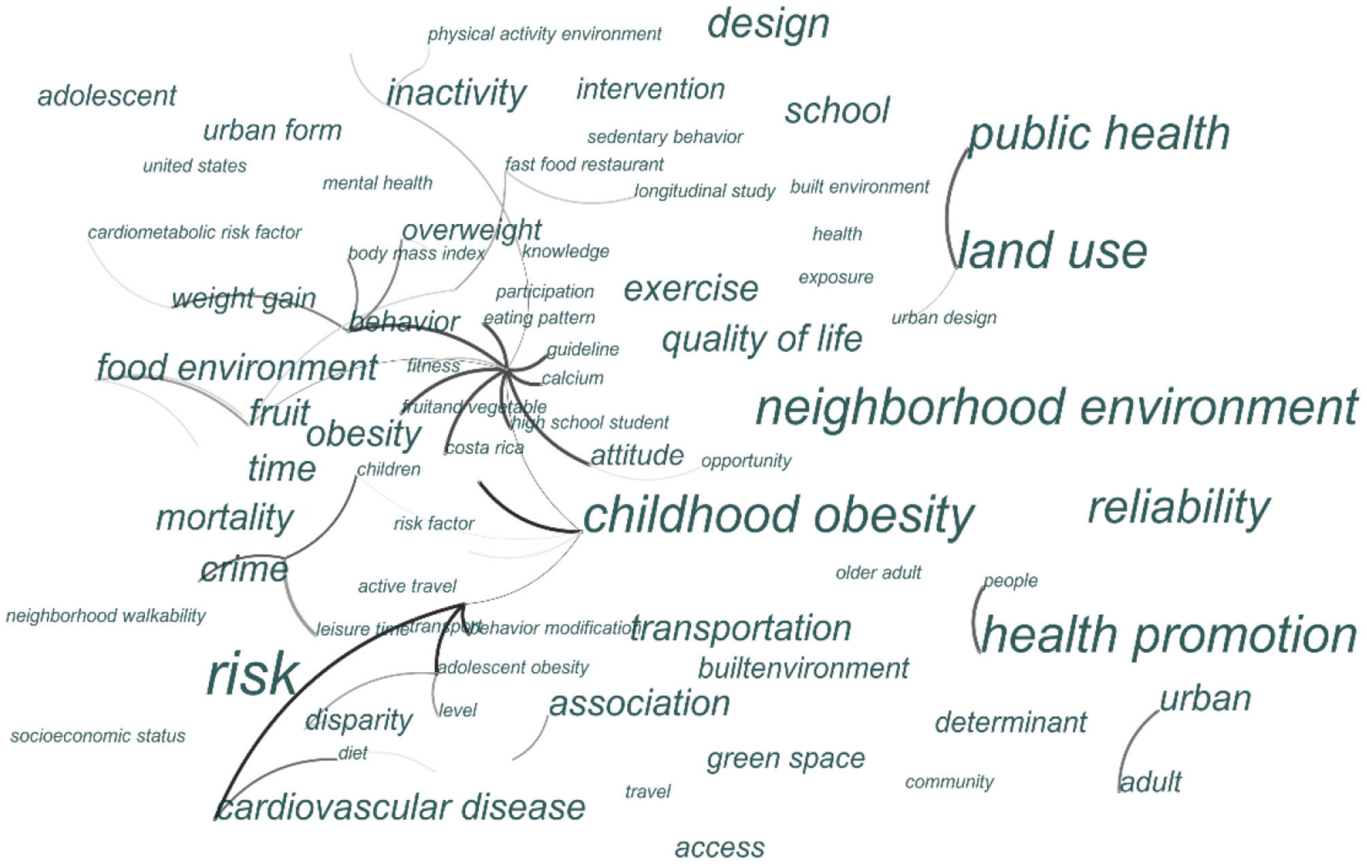
Cluster diagram of key issues related to the built environments and health.

**Figure 3:**
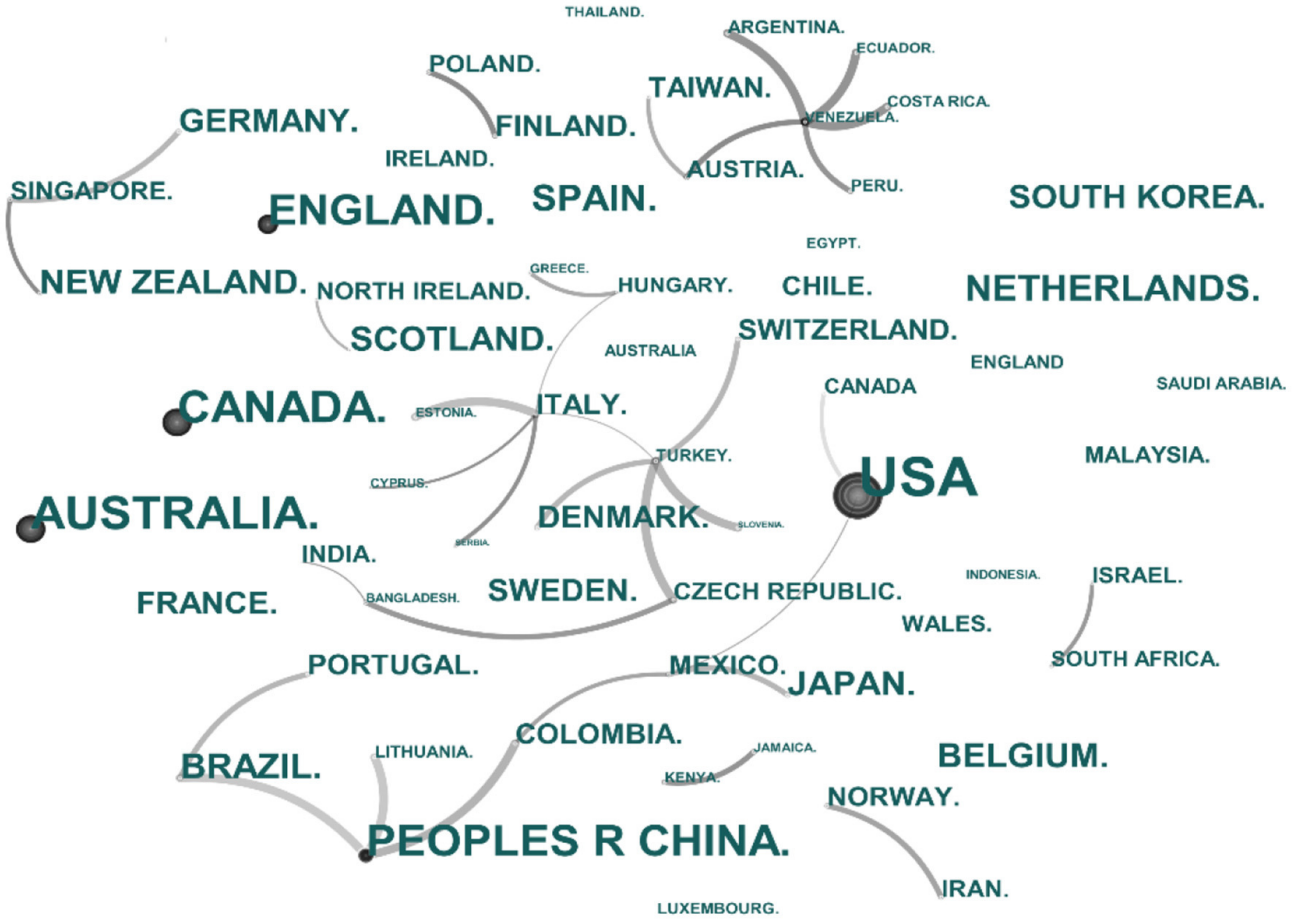
Cluster diagram of study areas related to the built environments and health.

With the increasing urbanization process and the transformation of urban environments, the impacts of various built environments on physical and psychological health have been a key area of interest for many scholars. Using the core data collection from the Web of Science (WOS) database as the literature source, a total of 3,750 articles were retrieved with the search criteria of built environments, physical activity, and health, spanning the period of 1995–2022. The application of Citespace was used to analyze the key issues and study areas of current literature. Figure 2 shows that current literature focuses on the effects of different factors in the built environments (BE) on an individual's health, such as land use, neighborhood environments, urban design, urban form, and transportation. In addition, body weight such as obesity, overweight, and childhood obesity is a widely-used important indicator of individual health conditions in current studies, and the effect of the built environment on body weight is one of the important hotspots in the related studies. As can be seen from Figure 3, those countries with high-level urbanization or under a rapid urbanization process pay more attention to examining the multifaceted impacts of built environments on public health, such as the United States, Canada, and China.

### Measuring the Built Environment and Health Outcomes

#### Ways to Measure Built Environments

Built environments compose of various physical environmental elements, such as land-use, transportation system, and other types of infrastructures. As one of the factors promoting transport-related and/or recreation-related physical activities and health gains, the built environment is an important aspect of public health-oriented urban planning. The characteristics of the built environments could be understood and examined from the perspective of “3Ds” (Density, Diversity, and Design) (22) and the extended “7Ds” features (Density, Diversity, Design, Destination accessibility, Distance to transit, Demand management, and Demographics) (23). The built environment that affects health is diversified, mainly including the physical activity environment, land use, and transportation environment, and the local food environments. For instance, it has been observed that a high level of physical activity is generally associated with a high level of mixed-land use, street or neighborhood connectivity, land development density, and neighborhood design (10).

Built environments have different categories, and there is a variety of indicators to measure built environments (Table 1). In general, owing to the development of location-based services and big data analysis, current literature has used more refined tools to measure built environments, physical activity, and health conditions, which helps understand the causality between these factors.

**Table 1:** Components and features of the built environments.

**Built environments**	**Main features and factors**
Land use	Green/open space
	Urban sprawl
	Land-use mix/diversity
	Land-use/population density
Transportation	Urban greenway
	Public transit stations
	Bike facilities
	Connectivity and accessibility
	Neighborhoods walkability
Service facilities	Food market supply
	Shops and stores supply
	Recreation facilities
Urban design	Urban or neighborhood design
	Urban renewal project
Community governance	Crime rate
	Seeing other activities

To measure the built environment's direct or indirect impacts on health, physical activity or the exposure to polluted environments have often been treated as mediating effects. Some studies have argued that the impacts of physical activity would be as least a part of mediating effect reflecting certain elements of built environments' effects on individual health condition (24). In fact, the mediating effect has been widely used in health studies to measure the relationship between physical activity (such as transportation-related or leisure-related travel behavior) and body mass index (BMI, such as for overweight or obesity)–a significant indicator of health condition (24). Indeed, the impacts of the various built environment on public health take effect through multiple forms, duration, and intensity of physical activities. Certain types of built environments can encourage or reduce active physical activity and thus have an impact on BMI and physical and mental health. Under a set of social rules, individuals would be shaped into certain particular patterns of activities they usually need. Long-time sedentary work, physical inactivity, and dependence on motor vehicles are among the causes of the rapid growth of chronic diseases (25). In particular, since the 1960s, the car-dominated mode of travel has led to an increase in the number and proportion of sedentary people, which has reduced physical activity and health conditions. Physical activity can significantly reduce the risk of obesity, cardiovascular disease, and other chronic diseases (26). For example, after controlling for variables such as age and hypertension, Frumkin et al. (27) found that the risk of diabetes in women who walked regularly was lower than those sedentary counterparts.

**Figure 4:**
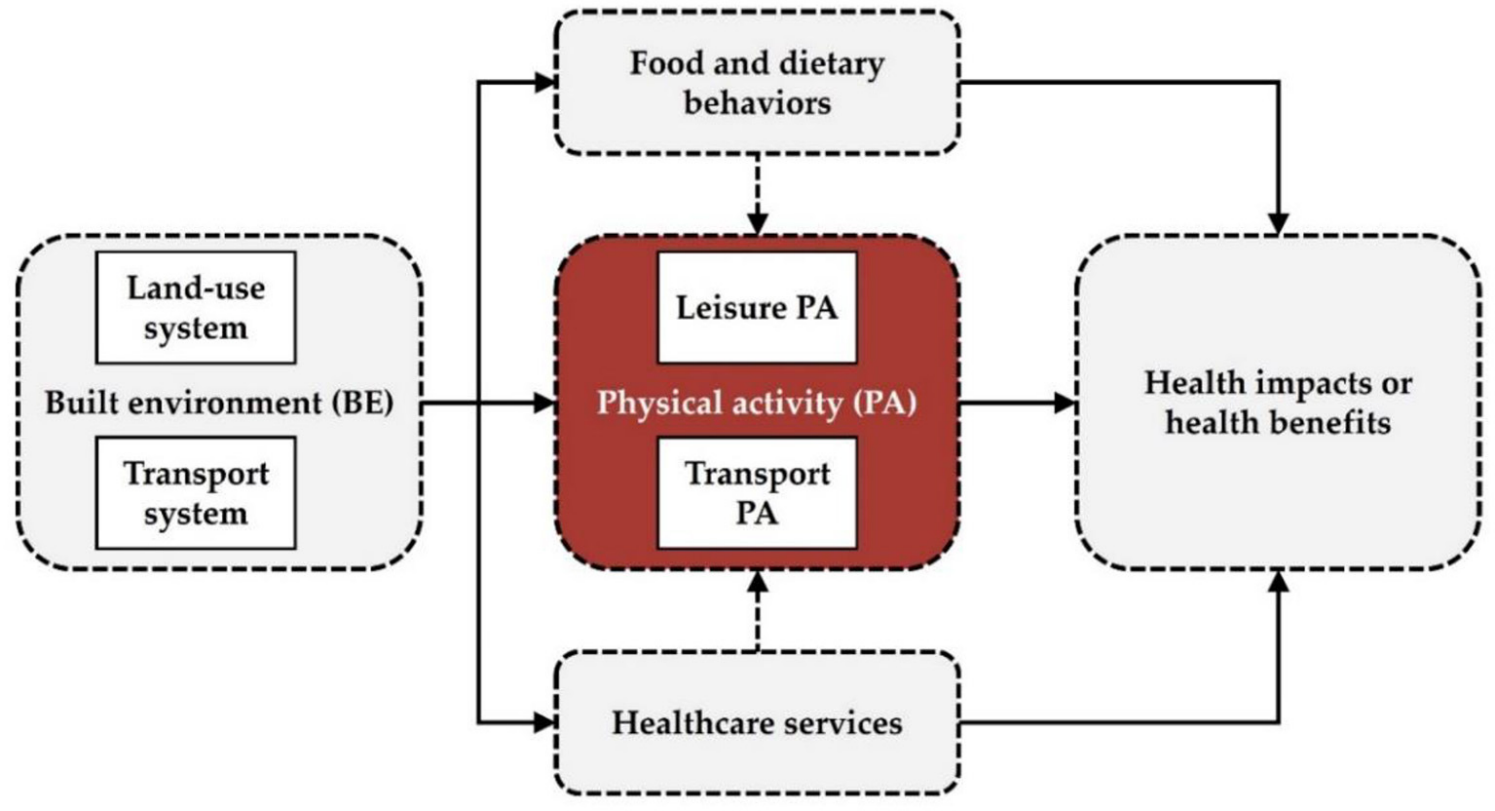
Overview of the built environment and health connection *via* physical activity.

Land use, population size, residential density, and infrastructure can significantly change individuals' travel behaviors and physical activities. Since the built environment is a geography-related issue consisting of a variety of components, there are several perspectives to understand built environments. Among these components, built environments could be measured in transport and land use aspects, which would significantly influence transport-related, and recreation-related physical activities, and dietary-related behaviors (Figure 4). Transport-related aspects of built environments include the provision of multi-modal transport services and the accessibility and connectivity to different locations for various travel purposes *via* transport services; while land use-related aspects of built environments include the provision of different land-use types, land development density, land use mixture, and land-use diversity.

#### Ways to Measure Physical Activities

Physical activities can significantly promote health benefits through energy-consumption and reducing sedentary and chronic diseases. The BMI and metabolic equivalent (MET) have been treated as instrumental variables to indirectly measure the health gains of physical activity (28, 29). To promote physical activity and to reduce obesity or overweight, governments at different levels have issued physical activity guidelines. For instance, the U.S. Department of Health and Human Services and the City of New York have, respectively released the Physical Activity Guidelines for Americans: Be Active, Healthy, and Happy (30) and Active Design Guidelines: Promoting Physical Activity and Health in Design (31). In addition to public sectors, the private sectors have also involved in promoting activity-friendly communities (32). These physical activity guidelines have provided a general set of recommendations on the amounts and types of daily needed physical activities, including moderate-intensity physical activity, vigorous-intensity physical activity, muscle-strengthening activity, and bone-strengthening activity (30, 33). Besides, multiply accelerometers, GPS devices or indirect calorimetry devices have currently been employed to track diversified forms and levels of physical activities; for instance, Fitbit and Fitbit Ultra are tested as one reliable and valid tool to monitor physical activity as well as to determine energy expenditure (12).

#### Approaches to Examining the Health Impacts of Built Environments

The diverse methods and devices to carefully examine the relationship between built environments and physical activity has gradually emerged. The development and advancement of data-sets, analytical techniques, and modeling methods have provided new perspectives and evidence for a more refined understanding of the complex relationship between the built environment and health benefits. First, in terms of data source and data set, the personal or household survey (34), interviews with specific questions (35), video and direct observations (36), and location-based big data (such as GPS tracking data) (37), which are cross-sectional, time-series, or (un)balanced panel data, have been widely used in empirical studies (Table 2). For example, the development of location-based technology and applications provides a new method to collect data on real-time travel and physical activities. The real-time data with fine temporal-spatial resolutions can be used to analyze and assess the daily, monthly, and annual energy consumption, which can well reflect the volume and frequency of physical activities. Second, the adoption of remote sensing (RS) and geographic information systems (GIS) techniques can help monitor built environment elements (38), such as land-use cover and change, population density and distribution, and air pollution distribution. There exist a series of quantitative models to examine the health impacts of built environments, such as the ordered logit model (34), quasi-experiments (10), before-and-after assessment (39), principal factor analysis (40), logistic regression model (15), binary logit models (41), geography-related regression (e.g., geographically weighted regression) (42), multilevel regression (3), and mixed-methods approach (43). Since some studies use aggregative data to examine the built environment—health outcome connections, the results would be misestimated if the issue of MAUP is not properly resolved (44). Besides, self-selection bias at the individual or neighborhood scale would occur in the case that some individuals tend to have a regular physical activity habitat but happen to live or work in a certain built environment. To cope with these issues, more objective-targeted models have been applied to avoid over-estimating or under-estimating the health effects of built environments.

**Table 2:** Measure and method of the built environment and public health.

**Classification**	**Details**
Data collection and dataset	The personal or household survey
	Interviews with specific questions
	Video and direct observations
	Location-based big data
Remote sensing and GIS tools	Land-use cover and change
	Population density and distribution
	Air pollution distribution
Models to examine the health	The ordered logit model
impacts of built environments	Quasi-experiments
	Before-and-after assessment
	Principal factor analysis
	Logistic regression model
	Binary logit models
	Geography-related regression
	Multilevel regression
	Mixed-methods approach

### Various Impacting Paths of Built Environments on Individuals' Health

The reasonable measurements of the built environment and physical activity are the basis of revealing the influence of built environments on physical activity and health conditions. On the one hand, the measurement of physical activity includes self-assessment reports, pedometers, and activity accelerator (45). The measurement of the built environment mainly relies on a questionnaire survey, quantitative statistics, qualitative assessment, and GIS spatial analysis. The built environment varies geographically across sites, communities, and regions. At the micro-level, the health impact of planning design can be qualitatively evaluated by obtaining detailed spatial data of built environments (including density, buffer, slope, and accessibility). At the macro level, the influences of the built environment on travel behavior, physical activity, and chronic disease were studied by statistical methods, including hierarchical modeling, sequential regression equation, and correlation analysis. For example, Kelly et al. (46) used data from the US health and nutrition survey to establish hierarchical modeling. They found that after controlling individual characteristics, regions with high accessibility and road network connectivity tend to show higher health levels (46).

The types, densities, and functional zoning of urban land use would significantly affect the spatial structure of communities and cities (Table 3). In general, the compact, high-density, and transit-oriented development (TOD) would better help guide physical activity and healthy lifestyles and then improve health conditions. On the contrary, the low-density land development pattern is more likely to have a lower rate of physical activity and health benefits compared to high densely populated urban areas (46). The density and mix degree of land development (53–55), as well as the proximity, connectivity, and accessibility of public facilities (68–70), can affect residents' travel demand (23), transportation mode choices (44, 74), and the frequency and intensity of physical activity (29, 46), thus influencing health conditions. For example, transport-related physical activity is positively associated with street connectivity, land development density, and land use mix (55). In other words, those areas with mixed land use and access to recreational and public transport facilities would increase physical activity. Besides, the impact of the built environment on health is also manifested at different spatial levels. At the node/site level, a built environment where people can be seen by more people when doing exercising is conducive to attracting residents to participate in relevant physical activities (35). At the community level, accessible green spaces and sports facilities, as well as street networks that facilitate non-motorized transportation, would benefit health *via* promoting social interactions and encouraging physical activity (47). Both the spatial and social aspects of built environments should be included to understand the associated impacts on physical activity and health. The crime rate is not a spatial pattern of built environments, but it is a very important social aspect of built environments that would influence physical activity and public health. Crime rate as an indicator of social security not only affects social governance and urban stability but also can generate negative effects on the physical and psychological health under a high-level crime rate.

**Table 3:** Multi-dimensional features of built environments affecting health conditions.

**Features of built environments**	**Physical activity**	**Dietary activity**	**Exposure to pollutions**
Green/open space	• (47, 48)		
Urban sprawl	⊚ (46)		• (49)
Urban greenway	• (50, 51); ⊚ (52)		
Public transit stations	• (34)		
Land-use mix/diversity	• (53–55)		
Land-use/population density	• (56, 57); ° (20)		
Food market supply	• (58, 59)	• (60, 61)	
Shops and stores supply	• (60, 61)	• (62)	
Bike facilities	• (63)		
Recreation facilities	• (64); ⊚ (65)		
Crime rate	° (66, 67)		
Seeing others active	• (35)		
Connectivity and accessibility	• (68–70)		
Neighborhoods walkability	• (57)		
Urban or neighborhood design	• (71, 72); ⊚ (65)		
Urban renewal project	• (73); ⊚ (52)		

*Source: compiled by authors based on relevant literature*.

*•, Positive impact; ⊚, No significant impact; °, Negative impact*.

Land use and urban transportation systems are highly interconnected. The built environment has an important influence on residents' commuting distance and the choice of transportation modes (75). Ewing et al. (23) pointed out that travel distance is generally affected by the characteristics of the built environment. In terms of transportation mode, those individuals who drive more and walk less would tend to have a higher obesity rate. For instance, by using the American Time Use Survey (ATUS) and dietary health data, Yang et al. (75) verified the relationship between travel behavior and body mass index from the perspective of time use and energy consumption balance, and found that prolonged car use can significantly increase the proportion of obesity, while non-motorized transportation can help reduce BMI and obesity (76). In the urban transportation system, green transportation such as walking and cycling has gradually become the focus of transport network design. Walking, in particular, a low-cost and convenient mode can be well-integrated into individuals' daily mobility. Vojnovic (77) found that neighborhood features such as short perceived distance and high connectivity can encourage residents to increase transport-related physical activity. It is worth noting that the relationship between built environments and travel behaviors and physical activities would be influenced by the issue of the individual- or neighborhood-level “self-selection” effects (10). Cao et al. (78) found that the built environment has a greater influence on travel behaviors than the self-selection effects.

### A Summary of the Built Environment—Health Outcome Connections

**Figure 5:**
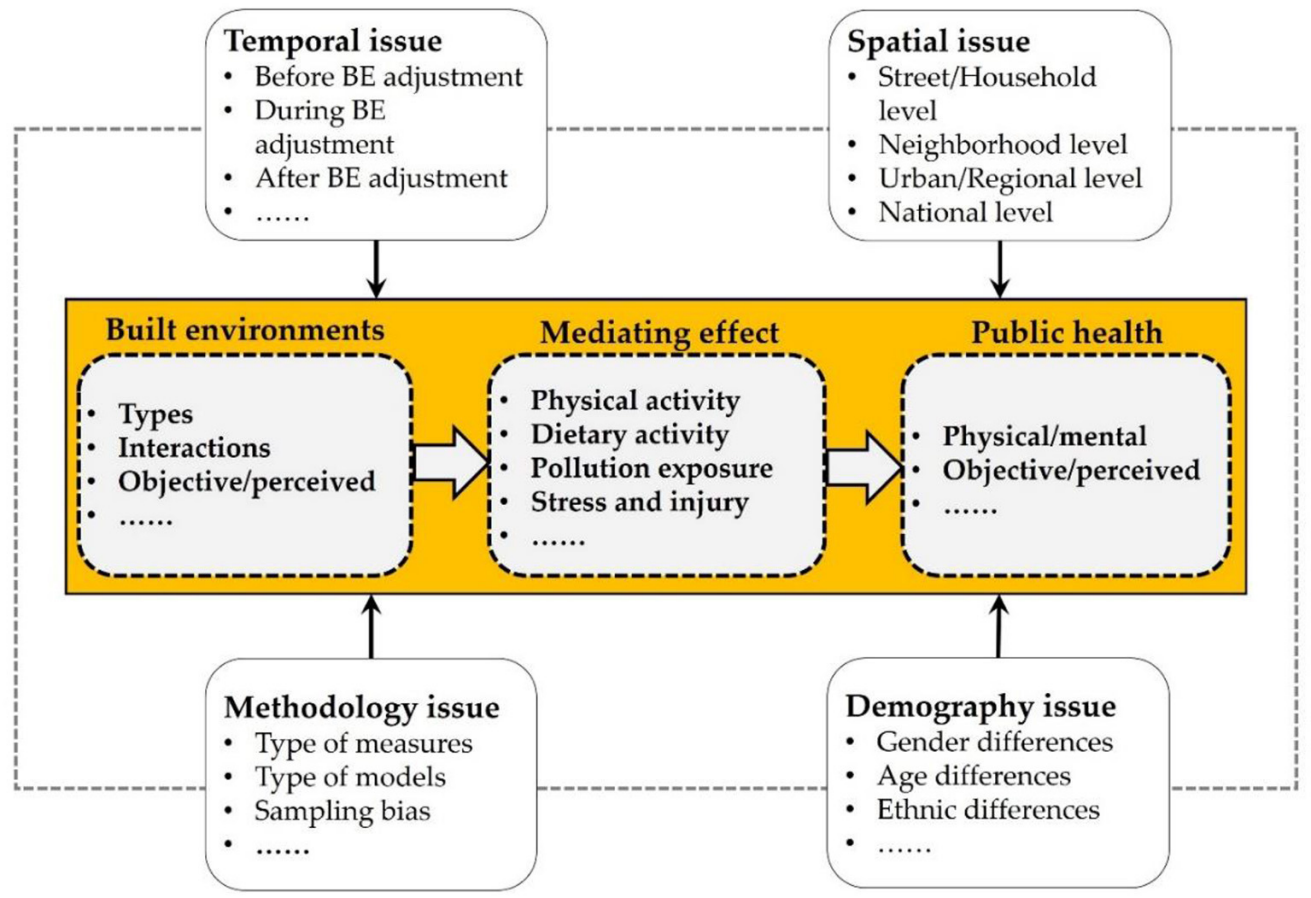
Significant issues that impact the built environment—health connections.

In general, there remains a quiet complex and dynamic relationship between the built environment and health outcomes and as a result, current literature does not reach a consensus on the built environment—health connections. How to measure these components would lead to different or even conflicting results and conclusions. In fact, according to current literature, several significant issues related to the time-span, geographical scale, research methodology, and demography would affect the results of the built environment—health relationships (Figure 5).

First, in the temporal aspect, the before-, during-, and after-adjustment built environments would have varied impacts on individuals' behaviors. Second, as one location is spatially correlated with another, the function of a particular element of the built environment would also be different under various spatial scales (i.e., street, household, neighborhood, urban, regional or national scales). Third, a variety of methodologies have been applied to examine the role of built environments in shaping individuals' physical activities or other types of behaviors, and some significant issues or concerns should well be addressed to get more precise results. For instance, Hong et al. (79) identified four major methodological problems leading to conflicting conclusions about the built environment—travel behavior relationship, which include “self-selection, spatial autocorrelation, inter-trip dependency, and geographic scale”. Currently, several methods have been gradually advanced to cope with these methodological problems. Last but not the least, the impacts of built environments on public health (physical vs. mental health, or objective vs. perceived health) would differ significantly across different social groups. Studies that examine the correlation or causation between built environments and public health should be careful of these issues.

Though multi-scale built environments can directly or indirectly generate impacts on individuals' physical activities as well as physical or mental health outcomes, some other important factors beyond built environments need to be considered. Otherwise, the relationship between the built environment and health outcome would be overestimated or underestimated. For example, due to the emerging technology developments, the adoption of health-related mobile applications would also change physical activities and eating habits, which can increase the added value of these mobile applications (80). Besides, even when the physical activity environment remains fixed, significant changes in food environments, such as changes in accessibility to healthy food stores, can still influence individuals' health conditions (4).

## Reshaping Built Environment's Impact on Health Benefits

During the large-scale urbanization, built environments have been significantly changed or intentionally reshaped. Some changes in built environments, such as the new provision of open green spaces, can undoubtedly help individuals to gain more health benefits through active physical activities. However, some transformation of the built environment would generate negative health benefits to some extent, such as the urban sprawls. Considering the health impacts of built environments at different geographical scales, it is quite necessary to understand the different impacts of built environments on different social groups and to adopt proactive planning or policy measures to avoid these negative externalities.

In the contexts of low-density development and urban sprawl, individuals' dependence on private vehicles would increase. As a result, the lack of physical activity and chronic diseases, including obesity and cardiovascular diseases, would become major risks of public health (10). Existing studies have advocated close connections between the built environment, travel behavior, physical activity, and public health. For example, the increase in car travel and the decrease in exercise time is more likely to lead to overweight or obesity (76). As a highly integrated and complex system, it is one of the important goals for urban planning to ensure the public health and sustainable development. In particular, when a large number of people live in large and high-density cities, public health would face severe challenges. Maantay (81) proposed that it is fully required to understand and evaluate the potential impacts of urban land development and infrastructure projects on public health. In general, according to the above-mentioned literature review and future urban and regional development trends, several planning and policy insights are required to promote public health and to reduce the health disparities among diversified social groups or geographical units as well.

First, considering the varied built environment conditions across different cities, it still needs to pay close attention to the temporal-spatial characteristics and strengthen the scientific research underlining the relationship between urban planning and public health, such as examining the impacts of the built environment on health conditions and the differences among populations or developing evidence-based health promotion planning and design. In particular, the urban planning authority should highlight the concept of public health and carry out effective measures to promote public health. For example, a health impact assessment (HIA) should be conducted in large-scale urban development projects. As a response to the emerging infectious diseases, local governments and planning authority should carry out reasonable programs to determine appropriate population size and its spatial distribution pattern, to determine appropriate land development density and the distribution of associated public services (such as the supermarket, open space, or leisure infrastructures), to directly or indirectly enhance the level of accessibility and convenience for individuals' physical activities. Additionally, according to the characteristics of disease transmission and health impact in a low- or high-density urban environment, the provision of multi-level medical service facilities should be strengthened, especially at the community level.

**Figure 6:**
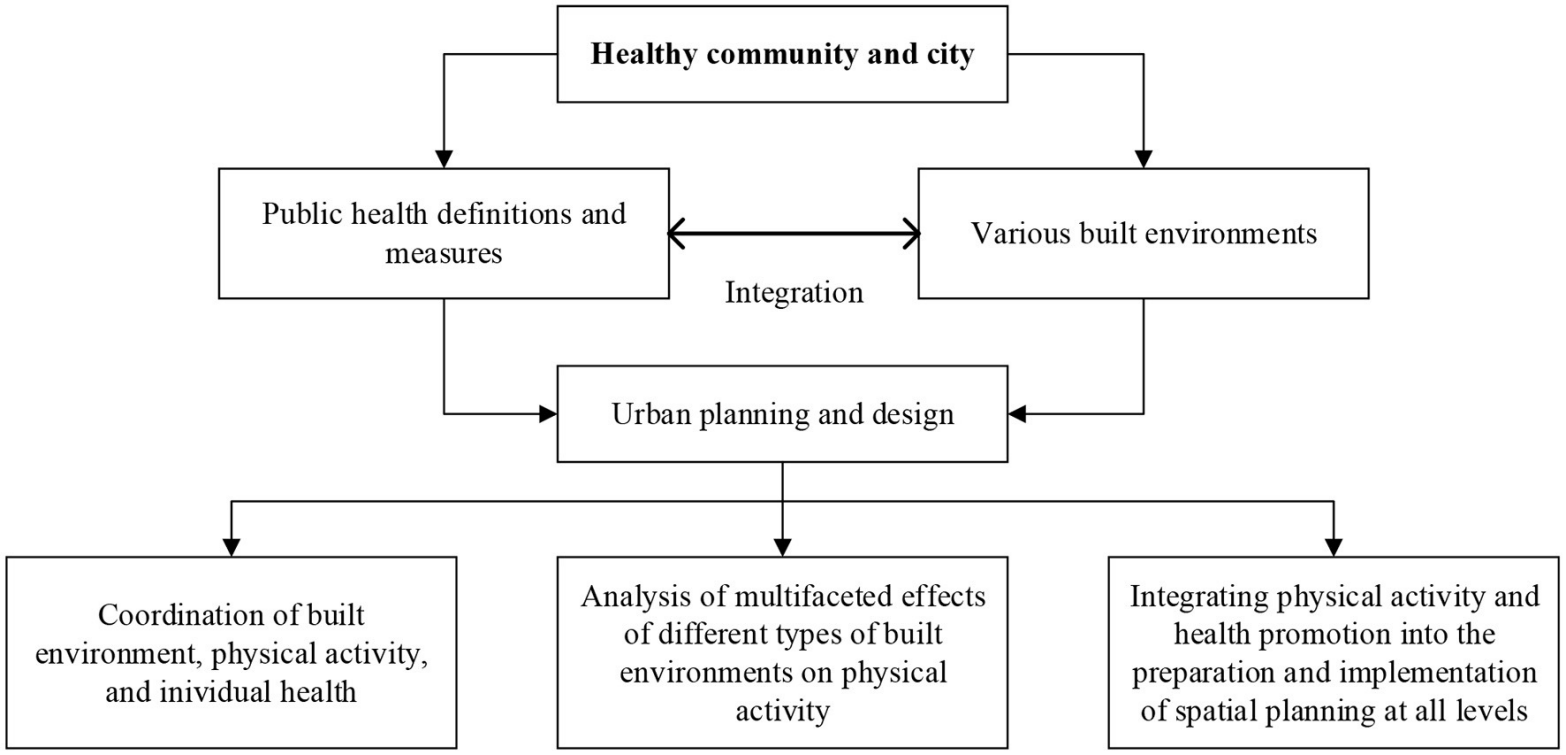
Integration of built environments and public health for a healthy community and city.

Second, the concept of public health is gradually incorporated into urban planning and micro-level urban design. The concept of “physical activity and health promotion” should be incorporated into multi-level spatial planning. First, from the perspective of mutual coordination of the built environment, physical activity, and health, the proposed standards or guidelines of physical activity (type, frequency, and intensity, etc.) for different groups should be highlighted. Second, the concept of physical activity and health promotion should be taken as a major objective in the preparation and implementation stages of all levels of spatial planning. The evaluation index system and evaluation scheme should also be objectively developed. Third, in terms of micro-level land use, transportation, and buildings, the effects of different types of built environments and their spatial combinations on physical activities need to be further studied. For example, it is needed to improve walkability in public space, green space, leisure, and retail commercial places to meet the daily physical activity needs of different groups (Figure 6).

Third, considering the various impacts of built environments on physical activity and health outcomes, it is of great significance to well plan and optimize the provision of public infrastructures and the associated public services. There are different development goals in the supply of public service under constrained budgets, such as equity, maximum coverage, or minimum cost (82). The connections between the built environment, physical activity, and health impacts have become an interdisciplinary issue, such as urban-rural planning, public health, and urban transportation. From this point, a further in-depth collaboration between healthcare, transport, and urban planning and design sectors are certainly needed (55). A well-designed policy and implementation framework for health-oriented planning and design as well as the provision of medical and health services would largely increase the health-related physical activities and corresponding health gains.

**Figure 7:**
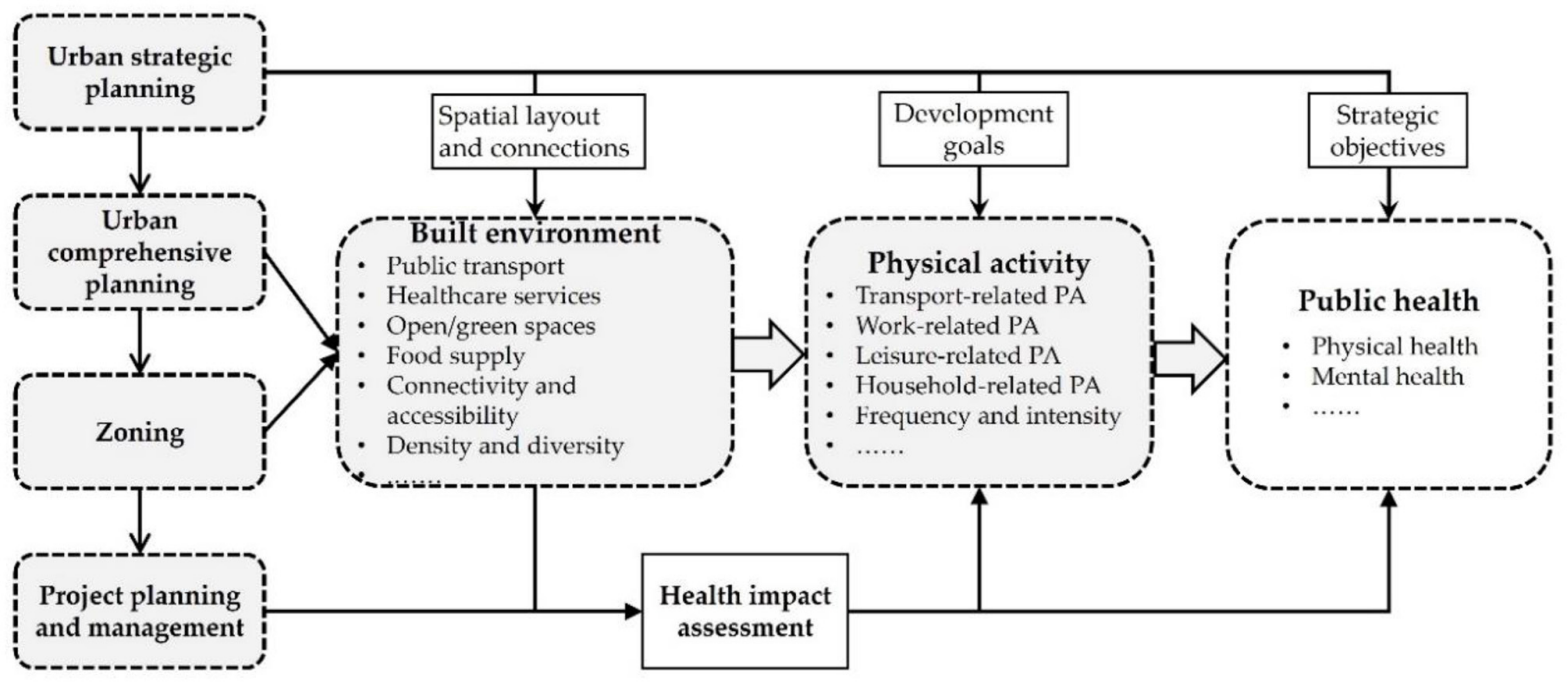
Health intervention in multi-level spatial planning.

Fourth, to understand the dynamics of major public health problems, society would benefit from regular health surveys and public health improvement plans. Regular surveys of individuals' health conditions and their medical needs will significantly improve our understanding of the status quo of public health. Besides, health impact assessment for major construction projects should be carried out, to identify possible impacts of project construction on public health, and to propose improvement measures (Figure 7).

## Conclusion and Discussion

During the rapid urbanization and globalization, there is a growing concern about promoting public health and facilitating the construction of a healthy city. Despite there are diversified paradigms to promote individuals' health gains, a growing body of evidence has highlighted that favorable built environments could generate significant and positive health outcomes through encouraging physical activities (83). Indeed, a better connection between built environments and physical activities has been considered a low-cost and effective way to improve health benefits (39). Research interests on the role of the built environment on physical activity and health benefits are growing, as individuals tend to suffer more from physical inactivity or be more exposed to various environmental pollutions under rapid (sub) urbanization. Current literature has used a variety of methods to examine the built environment–health connections from the perspective of physical activity and pollution exposure. This study conducts a systematic literature review on the built environment and health connections based on the published articles in the past decades. It also proposes potential policy insights for urban planning and development toward healthier cities. Certain built environment elements, such as the public transport stations, schools, sport and leisure facilities, or food markets, could be crucial for local governments or individuals to implement various forms of interventions aimed to promote healthy behaviors and health benefits.

Considering the complex influencing path and mechanism among built environment, physical activity, and health gains, urban planning and policies can significantly benefit from more researches on the built environment—health connections from the temporal-spatial perspective. First, the built environment—health connections would significantly vary across different spatial scales, such as the individual, household, neighborhood, district, city, or regional (metropolitan) levels. To address these issues, it is still of significance to examine how different built environment features would affect health benefits through multiple paths. Second, given that the reshaping of built environments would take time to bring effects, longitudinal experiments or before-and-after assessments examining temporal effect can better reveal how built environments can promote or reduce physical activity or health gains. For example, it would take time to observe newly built environment characteristics that bring effects on physical activity or health gains promotion (39). Third, the social aspects of built environments can also influence individuals' physical and mental health condition, and it is required to consider how the levels of physical activity and health condition of different social groups would be differential under built environments, which may generate social inequalities in health (84).

To plan and built a healthier city has become a core task of urban-rural planning in many cities or regions. Planning and policy decision-making would greatly benefit from the theoretical aspect of the built environment—health connections. First, since the public transit system is generally under-invested in many developing or even developed countries (85, 86), it is still necessary to give a high priority in the investment on the urban-rural transit system, which could promote active travel behavior and physical activity with low costs. Besides, individuals would have better access to public infrastructures (such as leisure, sports, and healthcare facilities). Second, as an individual's assessment of their own health status, the subjective health awareness refers to the degree to which individuals perceive their health status. Built environments would significantly affect individuals' subjective health awareness or objective health conditions. Individual's subjective health awareness is also a quite important indicator to reflect the impacts of built environments on public health. Implementing health-oriented planning and design and environment improvements would be helpful to promote physical or mental health conditions. For instance, small-scale environmental improvements would be effective to increase walking or cycling transport. Third, built environments are generally interconnected with social environments, such as social capital, social participation, or social cohesion, which can also affect individuals' physical and social activities. In particular, there is an association between mental health and social capital perceptions (trust and social cohesion) (87). Therefore, the associated social aspects of built environments and their impacts on health benefits should also be included in the health impact assessment.

In terms of the connection between the built environment and sports facilities, accessibility can be enhanced by integrating some trivial public spaces adjacent to green spaces in cities and improving the conditions of walking paths or bicycle facilities to enhance physical activity with a connection to transit services. For example, many cities in China have already deployed a large number of shared bicycles, which is one of the practices to enhance physical activity by improving cycling convenience. Regarding the risk of pollution exposure, from the sources of pollution, toxic and harmful gases and dust emitted from transportation and industrial production should be reduced, thus reducing the damage to the respiratory condition of the population. It is necessary to introduce certain incentives to promote new energy vehicles and public transit systems through giving some financial subsidies, which in turn would reduce the risk of pedestrian pollution exposure. For instance, municipalities can reduce car dependence by popularizing and optimizing public transport stations and routes, as well as by providing cheap, convenient, accessible, and efficient public transport services. In addition, more open/green spaces need to be planned and configurated in the built-up areas of cities to alleviate environmental pollution and promote physical activity. In order to cope with the physical and psychological attributes of public health among vulnerable social groups (e.g., children, women, and the elderly), it highlights the significance of incorporating the social and spatial aspects of built environments into the decision-making of policy and planning.

## Author Contributions

JZ: formal analysis, validation, and writing—review and editing. WL: formal analysis and writing—review and editing. BN: methodology and writing—review and editing. XL: conceptualization, formal analysis, methodology, supervision, funding acquisition, writing—original draft, and writing—review and editing. YD: conceptualization, formal analysis, methodology, and writing—review and editing. All authors contributed to the article and approved the submitted version.

## Funding

This study was supported by grants from the Key Project of Social Science Foundation of China (No. 20ZDA036) and the National Natural Science Foundation of China (No. 42001174).

## Conflict of Interest

The authors declare that the research was conducted in the absence of any commercial or financial relationships that could be construed as a potential conflict of interest.

## Publisher's Note

All claims expressed in this article are solely those of the authors and do not necessarily represent those of their affiliated organizations, or those of the publisher, the editors and the reviewers. Any product that may be evaluated in this article, or claim that may be made by its manufacturer, is not guaranteed or endorsed by the publisher.
